# Supplementary material for: Are carbon emissions trading and green financial instruments synergistic? -Comprehensive quantitative research based on content analysis
Source: PLoS One. 2024 Mar 7;19(3):e0298601. doi: 10.1371/journal.pone.0298601 (PMC10919601; doi:10.1371/journal.pone.0298601)
Supplement: S1 Data — (ZIP) [file pone.0298601.s001.zip › Data/地级市Z_F工作报告.zip!/║■─╧/╥μ╤⌠╩╨/└┤╫╘╣1⁄2╓┌║┼í╛┬φ┐╦╩2╛▌═°í┐_2005.docx]

各位代表：

现在，我代表市人民政府，向大会作政府工作报告，请予审议，并请各位政协委员和其他列席人员提出意见。

一、2004年政府工作回顾

2004年是新一届市人民政府履行职责的第一年。全市各级各部门认真贯彻“三个代表”重要思想和市第三次党代会精神，全面落实科学发展观，同心同德，积极进取，扎实推进各项工作，较好地完成了市三届人大一次会议确定的各项任务。全市完成生产总值287.33亿元，比上年增长11%，其中第一、二、三产业增加值分别增长8.2%、13.3%和10.8%。完成全社会固定资产投资75.71亿元，增长33.3%。实现社会消费品零售总额102.3亿元，增长13%。实际利用外资7317万美元，增长35%；实际利用内资42.1亿元，增长105.5%；进出口总额1.23亿美元，增长40%。完成财政总收入13.07亿元，增长14.87%，同口径比增长26.41%。城镇居民人均可支配收入8291元，增长11.7%；农民人均纯收入2713元，增长14.9%。城镇登记失业率控制在4.3%以内，人口自然增长率4.4‰。年末金融机构各项存款余额211.05亿元，贷款余额163亿元，比年初分别增长11.5%和11.3%。

工业经济规模扩大、活力增强、效益提高。完成工业增加值62.19亿元，增长12%。装备制造、棉麻纺织、食品加工、电力、造纸、建材等支柱产业产值大幅增长。国有企业改革成效显著。规模工业企业实现利润1.55亿元，比上年增加1.25亿元；工业经济效益综合指数达到105%，比上年提高15个百分点。企业技术改造取得成效，完成更新改造投资12.84亿元，增长24.2%，沅纸苇浆等重点技改项目顺利实施。益鑫泰衬衫荣获“中国名牌”称号，益阳橡机密炼机等13种产品荣获“湖南名牌”称号，滨湖柴油机等11家企业商标荣获“湖南省著名商标”称号。制订了2004—2020年益阳市产业发展规划。完成高新技术产业产值20.8亿元，增长13.8%。国家火炬计划益阳先进制造技术产业基地、湖南省高等院校科研院所成果转化及产业开发基地、深港产学研基地和中南大学科技园等“三基地一园区”落户益阳高新区。

农村经济产量增加、产值增加、收入增加。完成农林牧渔业总产值119亿元，增长8.4%。粮食总产190万吨，增长21.2%。出栏生猪、牛、羊分别增长6.1%、11.6%和11.2%，出笼家禽增长2.2%，水产品产量增长8.1%。完成杨树造林和退耕还林面积110.9万亩。劳务经济发展步伐加快，培训农民10.6万人次，桃花江保姆、益阳厨师、安化保安成为知名劳务品牌。农业产业化经营步伐加快，2家国家级龙头企业、9家省级龙头企业、39家市级龙头企业进一步做大做强。发展各类农民专业合作经济组织574个，入社（会）社（会）员15万多人，带动农户36万多户。农业综合机械化程度居全省领先地位。举办首届绿色农产品展销会取得圆满成功。全面贯彻落实党在农村的各项政策，广大农民在税费改革、粮食补贴、提高粮价中减负增收，直接受益。

招商引资、项目建设成效显著。坚持实施开放活市和项目立市战略，超额完成招商引资目标任务。成功引进了修山电站、东坪电站、柘溪电站扩容、泰格集团林浆纸一体化、319国道益阳南线高速公路、康利泰公司、兰溪米业新城、中南大学氧化锌晶须生产线、中天集团收购滨湖公司、湛江四通公司收购千山红糖厂等重点项目。成功举办了“湘洽会”益阳经贸活动，签约项目104个，合同引资80.27亿元，贸易成交额12.9亿元。与韩国南海郡签订了多项经贸合作协议，对外经贸合作空间进一步拓展。益沅一级公路、小马公路、白沙港和茅草街港千吨级码头、茅草街大桥、桃江资江大桥、通乡公路改造、通达工程等重点工程建设进展顺利。完成中心城区基础设施建设投资5.06亿元，建设了7条城市道路，亮化小街小巷22条，新增公共绿地40万平方米，改造、新建水冲式厕所138座，新置果皮箱1482个，新建密闭式垃圾围点100个，建成了团洲污水处理厂。完成小城镇基础设施建设投资4.8亿元，创建文明卫生城镇活动深入开展，城镇管理得到加强。完成水利建设投资8.59亿元，完成大堤培修198公里，填塘固基吹填105处，水库处险101处，新建山塘292口，改造电排机埠286个，气象、水文工作得到加强，防汛抗灾能力得到提高。通信基础网络不断改善，城乡电网改造基本完成。旅游基础设施建设力度加大，旅游业发展较快，完成综合收入11.06亿元，增长27.7%。房地产开发、商贸流通现代化建设步伐加快。

精神文明和民主法制建设取得新的成就，各项社会事业全面发展。深入进行以“三个代表”重要思想、树立和落实科学发展观为主要内容的学习教育活动，广泛宣传先进典型，大力弘扬社会正气和时代精神。切实加强未成年人思想道德教育。坚持“扫黄”、“打非”。继续开展十星级农户、五星级居民户等精神文明创建活动，促进了城乡文明程度和公民素质的提高。依法治市全面推进。认真执行人大及其常委会的决议、决定，自觉接受人大的法律监督、工作监督和政协的民主监督。共办理市人大代表建议、批评、意见169件，市政协委员提案222件，办复率100%。坚持依法行政，认真贯彻实施行政许可法，行政审批制度改革取得成效。法制宣传、法律服务工作力度进一步加大。群众测评机关作风活动取得实效，反腐倡廉、纠风治乱取得阶段性成果。安全生产事故明显下降。切实加强信访工作，深入开展反邪教斗争，严打整治工作成效明显，社会治安综合治理得到加强。深入开展食品、药品和农贸市场专项整治，打假治劣成效显著，市场经济秩序明显好转，经济发展环境不断改善。社会保障体系逐步完善，参保企业离退休人员基本养老金按时足额发放，养老保险、医疗保险和失业保险覆盖面扩大，城市低保、住房保障、扶贫开发、五保供养、农村特困救助、农村大病医疗救助工作得到加强，救灾救济工作有序进行，受灾群众和特困群体基本生活得到保障。解决了3万多名原国有农场职工的养老保险问题。住房公积金管理工作取得新的成绩。科技事业迅速发展，实施国家级科研项目6项，省级重点项目10项，申请专利130项。教育“普九”成果进一步巩固，高中阶段教育规模扩大，民办教育健康发展，素质教育得到加强。中心城区教育资源整合工作取得阶段性成果。湖南城市学院、益阳卫校新校区建设进展顺利，成功组建了益阳职业技术学院，益阳高级技工学校（筹）、湖南兵器工业高级技校（筹）均以高分通过了“创重升高”评估。全面完成年度人口计划，连续第14年获全省人口和计划生育工作先进单位。卫生基础设施建设取得突破性进展，全市开工建设的21个公共卫生项目全部完成，疾病预防控制体系进一步健全，血防体制改革全面完成。中国·益阳国家羽毛球队训练基地正式挂牌，做好了国家羽毛球队雅典奥运赛前来益封闭训练的接待服务工作，参加湖南省第二届大众运动会取得优异成绩。文化、广播电视事业有新的发展，文学创作日益繁荣，群众文化日趋活跃，撤地建市十周年联欢晚会等大型活动获得圆满成功。大力整治“网吧”违规经营和演出、出版市场，文化市场得到净化。耕地保护和土地开发利用工作有力推进，连续五年实现耕地占补平衡。加强了大气、水环境治理，认真开展了城区噪声污染防治工作，城市环境综合治理保持全省先进水平。统计工作被评为全国先进单位，做好了第一次经济普查的准备工作。国防动员、民兵预备役和“双拥”工作得到加强。审计监督工作力度加大。外事接待和出国（境）管理加强，对外交流合作不断扩大。台胞联络交往取得新进展，归侨侨眷权益得到保障。民族宗教政策得到落实，民族地区经济社会发展加快，宗教管理纳入法制化、规范化轨道。人防信息化建设、无线电管理工作得到加强。地方志编修工作进展顺利。档案管理利用和执法工作进入全省先进行列。地震预测和震灾预防体系逐步建立。残疾人事业稳步发展。其他各项工作都取得了新的成绩。

省市18件实事基本完成。去年，省为民办8件实事我市实施的项目，已通过省里的考核验收，全部完成和超额完成任务。新建通村公路275公里，列入洞庭湖区畅通工程的赤山大桥、麻河口大桥相继开工；企业基本养老保险新增参保人数4.2万人，失业保险新增参保人数1.1万人，医疗保险新增参保人数2.52万人，财政预算安排低保资金增长143%，企业基本养老金按时足额发放率达100%；为2.24万农村人口解决了饮水困难；新建沼气池2万个；新建城镇经济适用房14.2万平方米，改造乡镇敬老院20所，帮助345户贫困农户和80户城镇贫困居民户解决了住房。市政府承诺办好人民群众普遍关心和迫切需要办理的10件实事，除几件因国家政策调整和规划调整及情况发生变化外，其余均已完成或基本完成。①经省爱卫会组织专家验收组全面考核验收，我市已达到省级卫生城市标准。②城镇新增就业岗位3.47万个，农村劳动力转移达到82.4万人，均超额完成任务。③全市有213家市县属国有企业完成改革任务，占国有企业总数的56%。④高新区跨铁路高架桥因焊接受气候影响，调整至今年4月1日前通车，目前进展顺利；梅林路、云山路、迎宾路、团圆南路因国家关紧土地闸门未能如期开工，目前已拉通路基。银城南路、长春东路已建成通车。⑤8所农村中心卫生院改扩建或新建任务已经完成并投入使用，农村新型合作医疗试点工作年底已经启动。⑥完成县乡公路改造499.5公里，超额完成任务。因对路基沉降估计不足，益沅一级公路目前只完成了水泥底层铺设。马奎公路和沅茅公路均于2004年12月开工建设。由于省里的规划调整，桃马公路作为太澳高速公路联络线一并规划，南茅公路原设计单线后改为复线，两条公路暂未开工建设。⑦市委常委会议决定市一中由异地新建改为就地扩建，目前已完成规划设计和与益阳师范的合并工作。益师附小和市实验小学改造建设任务如期完成。⑧市特殊教育学校已建成使用，197名聋哑学生得到政府救助。⑨秀峰公园东大门、南大门、栖霞路建设任务基本完成。⑩天然气管道工程建设已按进度计划完成任务，今年7月1日将正式供气。

各位代表，过去的一年，我们遇到的困难比预料的多，工作成绩比预期的好，经济社会全面快速协调发展。回顾过去的一年，我们坚持发展第一要务，紧紧扭住经济建设这个中心，唱响加快发展的主旋律，聚精会神搞建设，一心一意谋发展；坚持自加压力，负重奋进，敢于攻坚克难，强力推进国有企业改革，突出抓好招商引资和项目建设；坚持以人为本，倾听群众呼声，关心群众生活，千方百计为群众排忧解难；坚持求真务实，大力改进工作作风，切实提高工作效率，狠抓各项工作的落实。成绩来之不易，这是坚持以科学发展观为指导，全面落实中央宏观调控政策和省委、省政府重大工作部署的结果，是中共益阳市委正确领导和市人大、市政协支持与监督的结果，是全市广大干部群众齐心协力、奋发图强的结果。在此，我代表市人民政府，向奋战在全市各条战线的工人、农民、知识分子、驻益人民解放军、武警官兵、公安干警，向支持益阳建设的海内外朋友，向关心益阳发展的同志们、朋友们表示崇高的敬意和衷心的感谢！

在肯定成绩的同时，我们也清醒地认识到我市经济和社会发展中还存在不少困难和问题。主要是：长期困扰我市经济发展的结构性矛盾，特别是工业化程度偏低的问题依然突出；经济总量不大，发展速度不快，经济外向度不高；经济运行质量偏低，财政困难，特别是县乡财政包袱较重；就业和再就业形势依然严峻，部分城乡居民生活困难；影响社会稳定的因素较多；发展环境有待进一步优化。对于前进中的问题，我们将通过加快发展切实加以解决。

二、2005年政府工作任务

（一）明确加快发展的指导思想和奋斗目标

2005年是“十五”计划的最后一年，是保持益阳经济社会发展良好势头、进一步加快发展的关键一年。做好今年的工作，对于全面实现“十五”计划目标，为“十一五”期间经济社会发展奠定坚实基础，具有十分重要的意义。我们必须万众一心，团结奋斗，努力加快经济社会发展步伐。

加快发展是益阳人民的迫切要求。发展才是硬道理。全国兴起了新一轮发展热潮，各地经济发展速度明显加快，呈现出万马奔腾、你追我赶的竞争态势。益阳的发展相对滞后，人民群众加快发展的愿望尤为迫切。我们必须顺应民心，切实保护好、引导好、发挥好各方面加快发展的积极性，形成谋发展、促发展、抓发展的强大合力，千方百计加快发展，通过加快发展增强市域经济实力，提高人民生活水平，实现强市富民的战略目标。

加快发展是益阳实现赶超目标的现实需要。益阳的主要问题仍然是发展不够。虽然近几年益阳的发展速度较快，但人家发展更快，去年我市生产总值增长率比全省平均水平低了1个百分点，与沿海发达地区、与省内发展较快的地区差距仍然很大。要缩小发展差距，实现后发赶超，与全国全省同步进入全面小康社会，我们必须奋发进取，顽强拼搏，以只争朝夕的精神，推动益阳经济社会快速发展。

加快发展是我们提高行政能力的具体体现。全市各级政府和各个部门一定要深刻地认识到实践“三个代表”重要思想，最根本的是要体现在加快发展上。发展是硬道理，必须大于一切；发展是第一要务，必须先于一切；发展是根本大计，必须重于一切；发展是解决一切问题的关键，必须高于一切。我们提高行政能力，必须首先提高领导加快发展的能力。要坚持把加快发展作为行政的首要任务，围绕加快发展制定行政的措施，用是否加快发展来检验行政的成效。

我们对加快益阳发展充满信心。当前益阳面临着加快发展的良好条件。世界经济呈现稳定增长趋势，国内经济将继续保持高速增长。国家加强和改善宏观调控，有利于我市更多地承接沿海发达地区的产业转移；实施中部崛起战略，将为我市加快发展带来新的机遇；继续加大对“三农”的支持力度，有利于我市农村经济加快发展。同时，我市经济发展基础逐步夯实，经济增长动力不断增强，经济发展速度明显加快。随着“三基地一园区”的落户、高新区南扩规划的实施，国企改革的不断深入、企业活力的不断增强、工业经济素质的不断提升，一大批基础设施与产业项目的开工建设和竣工投产，益阳将进入一个新的发展时期。只要我们抓住机遇，乘势而上，益阳经济社会发展就能跃上一个新台阶。我们一定要坚定信心，振奋精神，抢抓机遇，紧紧咬住发展不放松，坚持加快发展不动摇，努力加快益阳发展。

2005年政府工作的指导思想是：以邓小平理论和“三个代表”重要思想为指导，认真贯彻党的十六大和十六届三中、四中全会与市第三次党代会精神，全面落实科学发展观，坚持发展这个党执政兴国的第一要务，着力推进改革开放，继续调整经济结构，大力开展招商引资，突出抓好项目建设，做大做强工业经济，切实解决“三农”问题，努力实现经济社会全面快速协调可持续发展。

2005年的主要预期目标是：生产总值增长11%，全社会固定资产投资增长20%以上，社会消费品零售总额增长12%，财政总收入增长12%以上，城镇居民人均可支配收入增长8%，农民人均纯收入增长6%，新增城镇就业3万人，城镇登记失业率控制在5%以内，人口自然增长率控制在5‰以内。

（二）认真抓好加快发展的十项重点工作

1、把深化改革作为加快发展的强大动力来抓

继续抓好国有企业改革。坚定信心，下定决心，打好国有企业改革攻坚战。继续实行领导联系企业制度，充分发挥各级改革指导组的作用，主动为企业改革服务。围绕理顺企业产权关系和职工劳动关系，进一步优化方案，千方百计降低与筹措改革成本，严格执行市委、市政府一个《决定》、两个《实施办法》、一个《补充意见》，做好职工的安置和稳定工作。围绕盘活存量资产，进一步加大招商引资力度。采取转让、兼并、股份制改造、民营嫁接等多种形式，吸引更多的域外资金和民间资本参与国有企业改革。坚持以人为本的方针，努力解决改革中的各种矛盾和问题，维护职工合法权益，确保改革顺利推进。

切实抓好财政体制改革。以改善税收征管环境、理顺分配关系、促进财政收入增长为目标，以增强县乡财政实力、缓解县乡财政困难为重点，调整和完善财政管理体制。合理划分收入范围，完善转移支付办法。进一步深化收支两条线管理改革，推行国库集中收付和政府采购制度，合理确定支出次序，确保工资发放和机关正常运转，努力增加职工收入。继续抓好乡镇机构、农村义务教育和乡镇财政体制等综合配套改革，调动乡镇消赤减债和聚财、理财的积极性。

着力抓好政府机构改革和事业单位人事制度改革。认真实施省政府批准的我市新一轮政府机构改革方案。深化事业单位劳动人事、收入分配制度改革，引入竞争机制，实行人员聘用制、职称聘任制、结构工资制，大力精简人员，增强工作活力。对科技、教育、文化、卫生、体育等社会事业领域的经营性单位，逐步实行企业化管理。进一步完善国家公务员制度，推进国家公务员管理的法制化、规范化和科学化。

认真抓好以农村信用社为重点的要素市场改革，支持金融机构的改革和发展。进一步改革和完善投资体制，培育规范投资中介服务组织，广辟融资方式，拓宽融资渠道。建立健全人才市场、劳动力市场和技术市场，促进人才、技术、资金等各种生产要素的合理流动和优化配置。

大力发展民营经济。抓紧清理和修订不利于民营经济发展的政策，放宽市场准入，除法律、法规规定的外，取消所有前置审批。在投融资、税收、土地使用和对外贸易等方面，创造公平竞争环境。鼓励、支持民营企业以多种形式参与国有、集体企业改革。对已经改制的国有企业一律按民营企业对待，享受有关优惠政策。重点扶持市场前景好、发展潜力大、解决就业多、纳税多的民营企业发展壮大。

2、把招商引资作为加快发展的最佳选择来抓

继续开展招商引资年活动。调整招商引资方式，在全民招商的基础上，重点抓好专业招商、小分队招商、领导招商，努力实现招商引资在数量和质量上的突破，力争合同引资100亿元。明确招商引资的主攻方向，加强客户资源库建设，拓宽对外交往和项目对接的渠道。重点面向世界、全国500强企业开展招商，引进战略投资者，引进大项目。围绕产业发展规划，加大项目招商力度。精心组织一次规模较大的对外经济贸易洽谈会。强化各级各部门领导招商引资的责任。完善督办制度、目标管理制度、情况通报制度、年度考核制度、年终奖惩制度，健全重大项目洽谈机制、重点招商项目领导负责制，提高项目落户的成功率。加强投资项目全程代理服务工作，对外来投资项目实行分管领导和招商领导跟踪服务，切实提高服务质量，为投资商提供良好的创业环境。

3、把项目建设作为加快发展的重要途径来抓

抓好交通基础设施项目建设。尽快完成益沅一级公路、通乡公路等在建项目。加快东坪大桥、麻河口大桥、赤山大桥、马奎公路、沅茅公路、通村公路等项目的建设进度。尽快启动桃马公路、南茅公路、319国道益阳南线高速公路等项目的建设。

抓好工业项目建设。切实加强对新上工业项目的支持和服务，加强工程调度，力争早日竣工、投产、见效。重点抓好益阳电厂二期、柘溪电站扩容、修山电站、东坪电站、湘中木业、益阳橡机高等级子午胎设备研制生产基地等项目的建设。切实做好白竹洲电站、沅纸公司30万吨苇浆工程等项目的前期工作。结合国有企业改革，积极引进重大工业项目特别是装备制造业项目，逐步壮大产业规模。

抓好农业项目建设。切实抓好农业产业化、农业综合开发、移民开发和扶贫开发项目建设。抓好农村通信、用电、人畜饮水、广播电视等基础设施建设。

认真抓好“十一五”规划的编制工作。抓好项目库建设，根据国家的产业政策、投资方向和重点，按照全市产业发展规划，整理、谋划一批项目，不断完善项目库，做好项目整理推介工作，积极争取一批项目进入国家和省里的计划笼子。切实做好一批重大项目的前期工作。落实重大项目建设责任，强化工程监管，确保工程质量。

4、把工业经济作为加快发展的重中之重来抓

坚定不移地走工业强市之路，大力推进工业化进程，努力提高工业在三次产业中的比重，提高规模工业在工业中的比重。

大力培育支柱产业，重点建设一批标志性工程企业。坚持提升产业层次与扩大产业规模相结合，打造益阳工业经济的产业集群。以骨干企业为龙头，做大做强装备制造、棉麻纺织、食品加工、电力、造纸、建材等六大支柱产业，积极扶持高新技术、旅游两大新兴产业，改造提升化工、冶金两大传统产业。充分依托现有产业基础，选择一批有较大规模、广阔市场和发展潜力的高成长性企业，用2－3年时间，将其打造成为年销售收入过10亿元、税收过5000万元的标志性工程企业。

　　抓好企业的技术改造。把加快技术改造和技术创新作为改造提升传统产业的重要手段，按照专业集中、优质资源整合的要求，积极引导和扶持企业建立技术创新体系，走内涵扩大再生产的路子。积极争取国家、省有关部门对我市企业技改的资金扶持，确保今年全市完成技改投资12亿元以上。鼓励企业加大新产品开发力度，争创名牌产品，推动产品升级换代。以信息化带动工业化，提高企业应用信息技术的能力，增强工业的科技含量和核心竞争力。

引导园区快速健康发展。加快实施益阳高新技术产业开发区南扩规划，加强园区基础设施建设。以加快建设“三基地一园区”为契机，积极引进高新技术企业。引导资本、人才、技术等生产要素向园区聚集，把高新区打造成为益阳经济发展的示范区、产业开发的集聚区。区县（市）要加快发展园区经济，着力抓好区县（市）工业园和一批乡镇工业小区建设，引导中小型民营企业向园区集中，向“专、精、特、新”发展。

5、把解决“三农”问题作为加快发展的战略重点来抓

认真落实党在农村的各项政策。稳定农村家庭联产承包责任制，促进土地经营权合理流转和土地适度规模经营。进一步减轻农民负担。切实抓好粮食流通体制改革，认真执行中央和省里对种粮农民直接补贴、增加良种补贴和农机具购置补贴政策，认真落实农业税税率再降2个百分点和对安化县实行农业税全免的政策。加快农业科技服务体系建设，提高农业科技含量和农业机械化水平。加大农业执法力度，严厉打击坑农害农行为，切实维护农民利益。完善农户小额贷款制度，解决农民贷款难的问题。抓好全面小康示范村建设。

大力推进农业产业化。要用发展工业的理念来发展农业，积极推进农业产业化经营。支持农产品加工龙头企业的发展，扩大农产品基地规模，重点抓好200万亩生态型优质稻基地、120万亩优质双低油菜基地、100万亩优质棉麻基地、10万亩无公害蔬菜示范基地、5万亩有机茶和2万亩烤烟基地建设。因地制宜规划建设好瘦肉型猪基地、牛羊养殖带、家禽养殖区、网箱养鱼和特种水产基地，抓好大通湖渔场的综合开发。规范发展订单农业，加大市场开拓和网络体系建设力度，建设好农产品流通“绿色通道”。支持发展专业合作经济组织和行业协会，提高农民进入市场的组织化程度。

切实抓好农产品质量安全和畜禽防疫工作。大力发展无公害、绿色、有机农产品，加强农产品质量安全管理和认证工作，健全标准化生产体系，加快市农产品质量检验检测中心建设，切实抓好畜禽防疫、检疫工作，强化疫情监测和防控工作，确保养殖业健康发展和产品质量安全，增强农产品市场竞争力。认真办好第二届绿色农产品展销会，打造优质农产品销售平台。

大力发展“两南”林业。山丘区要高标准改造低产竹林20万亩，进一步抓好南竹加工企业的提质升级。平湖区要新植杨树100万亩，抓好泰格集团林浆纸、苏森公司林板材的基地建设。强化森林防虫、防火及林地林木管理工作，切实保护好林木资源。

积极发展劳务经济。把劳务输出作为改善农村就业、增加农民收入的有力措施来落实。切实加强农民工素质培训，继续着力打造桃花江保姆、益阳厨师、安化保安等劳务品牌。切实抓好维权服务，保障农民工合法权益。引导有资金、有技术的外出务工人员回乡发展农村二、三产业，促进农民就地转移。千方百计扩大劳务输出，转移农村劳动力稳定在80万人以上。

6、把城镇建设作为加快发展的重要载体来抓

加强城镇规划。坚持城乡协调、统筹发展的原则，高起点、高标准搞好城镇发展规划，充分发挥规划在城镇建设中的龙头作用。按照“文化古城、工业新城、休闲胜地、山水家园”的定位，完成中心城区总体规划和土地利用总体规划修编，编制好分区规划和详细规划，完善环境保护等专业规划和三个工业园区控制性详细规划，完成龙洲路、朝阳路及城区进出口和主要干道重要节点的景观规划，抓好梓山湖主题公园、秀峰公园后续工程等重要项目的规划。完善城镇体系规划，继续抓好县城和小城镇规划，推动县城提质扩容和小城镇规范发展。进一步强化规划管理。

加快城镇建设。突出抓好中心城区的建设，致力建设工业新城、打造生态绿城、构筑文化名城，增强中心城区的辐射带动功能。改造美化朝阳路，力争动工兴建资江四桥，加大中心城区排水主干管网改造力度，结合旧城改造抓好老居民区的设施配套。盘活城镇无形资产和存量资产，鼓励、引导社会资本参与市政公用设施建设，积极申请银行贷款，争取国家小城镇建设专项资金，加大城镇建设投入。积极支持13个重点镇加快建设步伐，完善城镇功能，走特色立镇、产业兴镇之路。

加强城镇管理。进一步完善城市管理办法，重点整治占道经营、乱摆乱放、乱搭乱建等行为。认真落实“门前三包”责任制，建立长效管理机制。巩固创建省级卫生城市的成果，启动国家级卫生城市的创建工作，通过2－3年的努力，达到国家级卫生城市的标准，进一步提升城市品位。加强社区建设，完善社区功能，着力加强对城中村的管理。强化部门协作，提高综合管理效能，形成城市管理工作合力。在中心城区推行相对集中行政处罚权制度，解决多头执法、重复执法、执法扰民等问题。创新城市管理体制，形成全民参与、齐抓共管的工作格局。深化户籍制度改革，推进户口城乡一元化管理，打开农民进城进镇落户的通道，只要有固定的住所，有比较稳定的收入，农民就可以落户城镇。加强小城镇管理，突出解决卫生状况差的问题。

7、把第三产业作为加快发展的重要增长点来抓

突出发展旅游业。立足益阳特色，整合旅游资源，启动创建“中国优秀旅游城市”工程，推动旅游业的发展。加强旅游基础设施建设，完成奥林匹克公园创国家4A级旅游景点工作。加强旅游文化建设，努力打造梓山湖主题公园、桃花江竹海、柘溪风景区、南洞庭湖湿地、农家乐休闲旅游等重点品牌。规范整顿旅游市场，提高旅游管理水平，优化旅游环境，吸引更多的国内外游客。成立国际旅行社，开展国际旅游服务。

加快发展服务业。运用现代经营方式和技术标准改造商贸流通、交通运输、仓储、餐饮娱乐等行业，不断提高传统服务业水平。发展社区服务业，引导居民增加体育健身和文化消费。加强对房地产业的规范引导和土地供给的宏观调控，着力打造住宅精品，提升人居品位，加强物业管理，搞好市场监管，促进房地产业健康发展。大力发展邮政、电信、网络、传媒等信息服务，提高金融、保险业和咨询、法律、会计等中介组织的服务水平。积极发展连锁经营、物流配送、电子商务和代理制，建立现代营销体系。

积极发展对外经济贸易。认真落实促进出口的各项政策措施，支持企业扩大出口。抓好出口企业及其产品的认证工作，大力推行国际标准，推动我市优势工业产品和绿色、特色产品打入国际市场。把扩大出口与优化进口结合起来，积极引进国外先进技术和设备，改造传统工业，提高产品的国际竞争力。实施“走出去”战略，鼓励有条件的企业跨出国门，开展产品展示和商品展销，促进技术和贸易交流。重点扶植益阳橡机、益华水产、科力远、鸿源稀土、惠同新材料、建玲竹业等一批出口规模大、竞争实力强、经济效益好的外贸企业。

8、把提高经济运行质量作为加快发展的主要目标来抓

着力提高财政收入质量。把增加财政收入、增强财政实力作为经济工作的出发点和落脚点，结合省以下财政体制调整后的税源结构和收入形势，制定新一轮财源建设规划，突出发展税收含量高的工业和财源增长潜力大、就业容量大的第三产业，形成财源效益型产业结构，努力提高财政收入占生产总值的比重。进一步调整和改革税收征管办法，不断完善税源监控机制，坚持依法治税，勤征细管，杜绝跑冒滴漏，做到应收尽收，防止虚增虚列、空转、转引税款的现象发生，努力提高税收收入占财政收入的比重。以省以下财政体制调整为契机，加大工作力度，使全市在新一轮体制调整中尽可能多地得到上级的支持，做大地方财政收入盘子，合理确定市县（区、市）乡（镇）财政体制，调动县乡两级培植财源的积极性，夯实地方财政收入持续稳定增长的基础，在做大税收规模的同时努力提高地方财政收入占财政总收入的比重。

切实转变经济增长方式。促进经济增长由粗放型向集约型转变，努力做到在节约资源中加快发展，在保护环境中加快发展，在发展循环经济中加快发展。加强矿产资源开发的规划和管理，提高市场准入门槛，严格限制一般性开采，严禁乱开滥采。着力发展深加工和精加工，延长产业链，提高产品附加值和资源利用率。积极推行清洁生产和节能技术，重点抓好冶金、化工、建材、造纸等行业节能节水的技术改造。实行高能耗、高物耗设备和产品的淘汰制度。关闭“五小”企业。按照统筹人与自然和谐发展的要求，更加重视工业污染防治和自然生态环境保护。加强建设项目的环境影响评价和环境监管，加大区域、流域污染治理力度。支持发展环保产业和循环经济，节约能源、节约用地、节约用材，实现低能耗、少污染、低代价、可持续的发展。

9、把社会事业作为加快发展的重要任务来抓

继续实施科教兴市战略。重点抓好“三基地一园区”的项目建设和科技攻关，加速科技成果的转化利用。争取7项科技项目进入国家科技计划笼子，15项进入省里的笼子。积极与深港产学研基地合作建设益阳产学研基地，搭建益阳高新技术孵化平台。搞好科普和科技培训工作。优先发展教育事业，依法加大对教育的投入。积极推进学校布局调整，抓好中心城区教育资源整合工作。进一步抓好中小学危房改造。继续巩固“普九”成果，全面抓好素质教育。大力发展职业教育、远程教育，支持高等院校加快发展，鼓励、规范社会力量办学。加强教师队伍建设，提高教师的师德和业务水平。落实国家资助贫困学生的政策和制度。实施人才强市。树立人才是第一资源的观念，制定和落实培养、引进、稳定和用好人才的政策措施。认真编制好“十一五”人才规划。广开招才引智渠道，大力吸纳省内外人才智力资源，加强企业家队伍建设，继续实施“农村适用人才工程”，为加快发展提供人才保证，营造有利于各类人才干事业、干成事业的社会氛围。

积极发展其他各项社会事业。推进文化体制创新，繁荣文学艺术、新闻出版、广播电视事业。加强基层群众文化活动阵地建设，动工建设市博物馆。办好《益阳日报》，扩大益阳广播电视覆盖面。加快疾病预防控制体系和医疗救治体系建设，加强传染病和血吸虫病的防治工作，提高公共卫生服务水平和突发性公共卫生事件应急能力。进一步加强农村卫生工作，搞好区域卫生规划，整合农村卫生资源，强化农村卫生基础设施建设。积极发展社区卫生服务。加强医德医风建设，提高服务水平。加强卫生监督体系建设，规范社会办医秩序。认真实施全民健身计划，组织好第七届大众运动会，积极备战湖南省第十届运动会，完善国家羽毛球队训练基地配套设施，发展体育产业。认真贯彻实施《妇女儿童发展纲要》。全面落实计划生育基本国策，继续实行计划生育一票否决制，完善人口和计划生育目标管理责任制，抓好农村计划生育家庭奖励扶助试点工作，控制人口数量，提高人口素质，改善出生人口结构，进一步稳定低生育水平。认真抓好第一次经济普查工作，确保普查数据质量。深入实施食品药品放心工程，推行食品市场准入制，抓好农村药品监督和供应两个网络建设，维护人民群众的身体健康和生命安全。强化国防教育，提高国防意识，广泛开展拥军优属、拥政爱民和军民共建活动，创建“双拥”模范城，支持部队建设和民兵预备役建设。加强人民防空工作。继续做好民族、宗教、外事、侨务、对台、气象、水文、档案、老龄、地方志等工作，进一步发展其他各项社会事业。

10、把加强民主法制建设和精神文明建设作为加快发展的保障措施来抓

加强民主法制建设。进一步增强民主法制观念，坚持向人大及其常委会报告工作和向政协通报工作的制度，切实执行人大及其常委会的决议、决定，广泛听取各民主党派、工商联和无党派人士的意见，认真办理人大代表建议和政协委员提案，自觉接受人大的法律监督、工作监督和政协的民主监督。积极支持工会、共青团、妇联等人民团体的工作。加强基层民主政治建设，认真做好村民委员会第六次换届选举工作，全面推行政务、厂务、村务公开，切实保障广大人民群众的民主权利。大力推进依法治市，继续抓好“四五”普法教育，进一步提高领导干部、执法人员和青少年的法律素质。认真贯彻落实《全面推进依法行政实施纲要》，切实提高依法行政水平。深入贯彻实施行政许可法，进一步推进行政审批制度改革。加强执法队伍建设。加强行政执法监督，严格落实行政执法责任制和执法过错追究制，做好行政复议、行政应诉等工作，把行政行为纳入法制化轨道。

加强精神文明建设。认真贯彻落实《公民道德建设实施纲要》，以弘扬爱国主义和民族精神为核心，加强社会公德、职业道德和家庭美德教育，提高公民道德素质。破除陈规陋习，倡导文明健康的生活方式。加大对“网吧”的整治力度，积极推进村级电子阅览室建设。坚持不懈地开展“扫黄”、“打非”行动，净化文化市场和社会风气。继续广泛开展农村创十星级农户、城镇创五星级居民户等主题鲜明、丰富多彩的群众性精神文明创建活动，推动各行业文明服务和城乡良好社会风尚的形成。

（三）着力解决影响加快发展的六个问题

1、增强防汛抗灾能力，确保安全度汛。把防汛抗灾摆在十分重要的位置，做好防大汛的各种准备。进一步加大水利建设力度，继续抓好湖区险工险段的处理、内涝治理、山区病险水库的除险保安和资江整治工作，全面完成一线防洪大堤两水夹堤堤段的填塘固基吹填任务，认真做好避、防、抗、救山洪灾害的工作，切实加快城市防洪工程建设，不断提高我市防洪保安能力。

2、高度重视安全生产，保障人民群众生命财产安全。牢固树立抓生产必须抓安全的理念，严格落实安全生产行政首长负责制和责任追究制，加大安全生产投入，加强安全生产目标管理，深入开展经常性的安全生产大检查，进一步开展安全生产专项整治和安全隐患整改，依法抓好安全生产许可证的颁发，严厉打击非法生产，有效防止重特大事故发生，实现安全生产事故、死亡人数、重伤人数、直接经济损失等几大指标明显下降。

3、加强社会治安综合治理，确保社会政治大局稳定。坚持社会治安综合治理一票否决制，落实责任追究制。建立和完善治安防控体系，促进社会治安状况持续好转，创建“平安益阳”。严厉打击地下“六合彩”等赌博活动和“法轮功”等邪教组织，严厉打击各种严重刑事犯罪和经济犯罪。高度重视处理新形势下的人民内部矛盾，坚持“分级负责、归口办理、属地管理”的原则，全面落实信访工作责任制，综合运用法律、政策、经济、行政等手段和教育、协商、调解等方法，依法及时、有效地处理群众来信来访，加强人民调解工作，将各类不稳定因素化解在萌芽状态。强化基层化解各类矛盾的能力，及时妥善处置各种群体性、突发性事件。

4、突破资金、土地瓶颈制约，支撑经济快速发展。深入开展金融安全区、诚实守信区和企业征信体系建设，努力营造诚信的社会信用环境。促进银企合作，引导金融机构增加信贷投入，优化信贷结构，支持经济发展。加强金融监管，维护金融秩序。继续抓好“两会一部”、城市信用社的清收兑付工作。千方百计解决用地不足的矛盾。积极向上申报，争取更多的用地计划指标；盘活土地存量，依法收回闲置土地，确保建设用地供应；大胆探索解决用地问题的新办法，引导集体经济组织依法以土地入股和出租参与项目建设。

5、突出关注民生，努力为群众办实事。切实抓好就业再就业工作，完善就业服务体系，搞好就业培训和指导，落实再就业优惠政策，千方百计增加就业岗位。进一步完善城镇企业职工养老、医疗、失业保险制度，扩大各类社会保险覆盖面，继续巩固“两个确保”，全面启动工伤、生育保险，做好城市低保提标工作。积极做好“双联”、扶贫帮困、灾民安置、残疾人等工作，建立健全社会救助体系。加大工程款、农民工工资和征地拆迁补偿款的清欠力度。认真解决群众关注的热点、难点问题，切实解决群众的实际困难。在抓好去年10件实事后续工作的同时，今年办好关系人民群众切身利益的10件实事。①完成无公害、绿色农产品认证50个，产地认定面积100万亩。②茅草街大桥、桃江资江大桥建成通车，茅草街港和白沙港千吨级码头竣工投入使用。③完成20所乡镇敬老院的扩改建任务。④动工兴建中心医院门诊大楼，重点支持10所农村卫生院建设，搞好桃江县新型农村合作医疗制度试点。⑤帮助1万人越过低收入线。⑥在中心城区支持搞好10个社区建设。⑦完成秀峰公园三期工程建设，启动朝阳路改造提质工程，拉通改造好10条小街小巷，加快高新区道路畅通工程建设。⑧全面完成天然气利用工程。⑨基本完成国有企业改革任务，城镇新增3万人就业。⑩完成中心城区户牌、街牌、路牌设置工作。

6、改进政府机关作风，提高政府工作效率。大力加强政风建设。按照为民、务实、清廉的要求，强化宗旨意识和服务观念，坚持深入基层、深入群众，真心实意为群众办实事、做好事、解难事。坚持求真务实，真抓实干，不搞形式主义，不做表面文章，不贪图虚名。牢记“两个务必”，弘扬艰苦奋斗精神，大兴勤俭节约之风。全面实施《建立健全教育、制度、监督并重的惩治和预防腐败体系实施纲要》，认真贯彻落实中央、省委关于领导干部廉洁自律的十六条规定，严厉整治不正之风，严肃查处腐败案件。完善规章制度，加强行政监察和审计监督，强化对各类财政性、政策性资金的监管。改革目标管理考核办法，对各区县（市）的工作主要考核六大经济指标，对市直部门的工作既考核业务工作，又看其为加快益阳发展所作贡献的大小。坚持不懈地优化经济发展环境。继续把优化经济发展环境作为“一号工程”来抓，全面推行分级负责制、部门首长负责制和责任追究制，完善奖惩办法，落实主要责任人的责任，切实解决政令不通、效率不高、服务不优的问题。继续开展群众测评机关作风活动，认真落实同市一家检查制度、行政审批限时办结制度、首查先行责令改正制度、首问责任制度、挂牌上岗制度、经济环境110制度、典型案件通报和黄牌警告制度。加强对企业发展环境和重点工程施工环境的整治，严厉禁止各种乱收费、乱罚款、乱摊派行为，严厉打击各种强揽工程、强行阻工、敲诈勒索、偷盗哄抢等行为。努力提高政府工作效率。在全市各级政府机关普遍开展“提高办事效率，为加快发展作贡献”的教育活动。全面完成政务中心建设，除法律法规有明确规定的外，所有行政审批事项一律进入中心办理，实行窗口受理、并联审批、限时办结、统一收费。进一步改进政府工作流程，简化办事程序，治理“文山会海”，减少文件、会议。加快电子政务建设步伐，积极推行网上办公。加大行政督查力度，狠抓工作落实，确保政令畅通。

各位代表！我们正处在加快发展、实现赶超的关键时期，新的形势催人奋进，新的任务光荣艰巨。让我们高举邓小平理论和“三个代表”重要思想伟大旗帜，紧密团结在以胡锦涛同志为总书记的党中央周围，在中共益阳市委的领导下，同心同德，开拓进取，自加压力，负重奋进，为圆满完成今年的各项任务，推进益阳全面建设小康社会的伟大进程而努力奋斗！
